# Experiences, perspectives and priorities of people with schizophrenia spectrum disorders regarding sleep disturbance and its treatment: a qualitative study

**DOI:** 10.1186/s12888-017-1329-8

**Published:** 2017-05-02

**Authors:** Sophie Faulkner, Penny Bee

**Affiliations:** 1 The School of Health Sciences, Oxford Road, Manchester, M13 9PL UK; 2 Greater Manchester Mental Health NHS Foundation Trust, Trust Headquarters, Bury New Road, Prestwich, Manchester, M25 3BL UK

**Keywords:** Sleep, Insomnia, Circadian rhythm disorder, Schizophrenia, Psychosis, Antipsychotic, Daytime functioning, Qualitative, Interpretive phenomenological analysis, Interview

## Abstract

**Background:**

Sleep problems are very common in people with schizophrenia spectrum disorders, and impact negatively on functioning and wellbeing. Research regarding interventions to improve sleep in this population has been lacking. Little is known regarding these patient’s perspectives on sleep problems and their treatment, providing very little foundation on which to develop acceptable and patient-centred treatments.

**Methods:**

This study aims to explore perspectives and priorities of participants with schizophrenia spectrum disorders regarding sleep and sleep disturbance, and their perspectives on existing treatments. An Interpretive Phenomenological Analysis (IPA) study was conducted; data were gathered through in depth interviews with 15 people with schizophrenia spectrum disorders and varying degrees of self-reported sleep disturbance, each case was analysed individually before cross-case comparisons were made.

**Results:**

Sleep maintenance and sleep quality were universally valued. Changes to sleep were interpreted as part of a perceived loss of normality relating to diagnosis. Participants differed in the extent of any hopes that sleep would improve. Sleep disturbances were linked to a reduced ability or opportunity to participate in valued activities, and were entangled with self-image due to a wish to be perceived as alert and in control. During difficult times, sleep could be seen as an escape. Concerns were expressed regarding the negative effects of using hypnotics or anti-psychotics to aid sleep, although typically antipsychotics were deemed more acceptable than hypnotics. Concerns regarding barriers to adherence and effectiveness of self-help approaches were common. Non-pharmacological interventions were noted to require a personalised whole-lifestyle approach.

**Conclusions:**

This is the first study to explore sleep perspectives in participants with established schizophrenia spectrum disorders, recruited from a population receiving usual care. Findings re-enforce the importance of considering sleep within recovery focused practice. In developing and adapting interventions routine-based approaches should be considered. Approaches should attempt to make gradual changes more easily perceptible, should support motivation for behaviour change, and should consider the impact of regular psychotropic medications.

**Electronic supplementary material:**

The online version of this article (doi:10.1186/s12888-017-1329-8) contains supplementary material, which is available to authorized users.

## Background

Sleep disturbances are much more common in people with serious mental illness than in the general population [1], and the negative effects of poor sleep on recovery in a range of psychiatric conditions are well-established [2]. Although not always cited as a core symptom of psychotic illnesses [3], sleep disturbance is present in up to 80% of people with schizophrenia [4]. Sleep disturbance is highly prevalent in the early course of the condition prior to the emergence of psychotic symptoms [5], and often persists after other symptoms have been treated [6].

Knowledge regarding treatment of sleep disturbances in the general population has recently been applied to populations with serious mental illness. Early indications suggest Cognitive Behavioural Therapy for Insomnia (CBT-i) is effective in patients with co-morbid depression, anxiety, Post-Traumatic Stress Disorder (PTSD), and substance abuse [2]. Treatment of sleep problems in people with schizophrenia has been comparatively neglected [7]. A case series has suggested CBT-i reduces insomnia, and reduces persecutory delusions [8], and a larger pilot study (conducted by the same research group) has found evidence of improvements in insomnia, although this study did not evidence an effect on persecutory delusions or hallucinations [9].

Management of sleep disturbance is neglected in clinical guidance regarding psychotic illnesses when compared to other mental illnesses. Sleep is discussed in guidance regarding depression [10] and PTSD [11], but is notably absent from guidance for psychosis with coexisting substance misuse [12], and psychosis and schizophrenia [13]. Monitoring for co-existing conditions, and comprehensive assessment of physical, social, occupational, and quality of life related factors is advised, but sleep is not explicitly mentioned.

Poorer sleep is associated with reduced quality of life [14] and increased suicide [15] in this population. Evidence suggests circadian dysregulation contributes toward the cognitive impairment associated with schizophrenia [16, 17], and poorer subjective and objective sleep has been shown to predict worse next day functioning and symptoms [18], suggesting sleep as an important treatment target.

Circadian rhythm disorders are more common in schizophrenia than in poor sleepers without psychiatric co-morbidity, or in other serious mental illnesses [19]. Findings suggest a reduced effect of melatonin in this population [20], and altered sensitivity to environmental cues [21]. It is now acknowledged that circadian dysregulation in this population should not be attributed solely to the effects of medication and lifestyle [19]. Studies have shown alterations to sleep architecture in people with schizophrenia, different to those found in depression or primary insomnia, suggesting the mechanisms causing sleep disturbance may also vary between these populations [22]. Therefore it is likely that sleep experiences may also be distinctive in this population.

It is important to consider patient perspectives in the design of non-pharmacological sleep interventions, including pre-treatment perspectives, in order to understand barriers to seeking treatment, or adherence to treatment. A systematic review found only two qualitative studies examining the perspectives of people with schizophrenia spectrum disorders regarding sleep treatment [23]. One recruited patients from the treatment arm of a pilot trial of CBT-i [24]. The other conducted focus groups examining views on three major evidence based sleep treatments (hypnotics, CBT-i, melatonin), but did not explore sleep experiences [25]. The present study represents the first to explore perspectives on sleep disturbance in people with longstanding schizophrenia spectrum disorders, in a sample who have not received specialist psychological sleep treatment.

## Methods

### Aims

As described, sleep disturbance is highly prevalent in people with schizophrenia spectrum disorders. Sleep impacts on quality of life, functioning and recovery, yet there is limited evidence regarding the experiences, beliefs and priorities of this population in relation to sleep, its assessment and treatment. The present study set out with the following objectives:

- To explore expectations and priorities regarding acceptable, optimal, and current sleep.
- To elicit and examine personal explanations in relation to sleep; what causal factors people believe contribute to sleep quality and sleep need, and what is attributed as being an effect of good or poor sleep.
- To explore people’s experiences or expectations of a variety of intervention approaches, identifying factors which contribute toward perception of an intervention as effective, appealing or achievable.

### Sample

The target sample was 8–15 participants; slightly larger than average for an Interpretive Phenomenological Analysis (IPA) study [26]. This was for three reasons: Firstly, to maintain anonymity it was deemed inappropriate to present a single case or few cases in detail [27]. Secondly, a somewhat diverse range of sleep disturbances were to be included, requiring more data for an understanding to be reached [28]. Thirdly, it was anticipated that some participants who chronically suffer negative symptoms of psychosis, may be less able to provide rich data on all topics discussed.

Some qualitative researchers advocate seeking out ‘good informants’ who are ‘knowledgeable’ on the topic, ‘reflective’ (p127) [29], and can provide richer data [30]. However it was felt that it was important to access the experiences of people who suffer chronic symptoms and impairments, not just those who are more recovered.

A small, yet experientially representative sample [31] was desired. Maximum variation [32], or purposive, sampling was used; seeking participants with a range of characteristics which might influence their contribution [30]. A range of severity and nature of sleep timing, initiation and maintenance issues were sought, as were participants with a range of levels of functioning: from those with varied occupational lives and good social networks, to those who were occupationally deprived and isolated. This was achieved through discussions with gatekeepers, and by using varied recruitment methods to reach participants with different characteristics [33].

### Inclusion and exclusion criteria

Inclusion criteria were: 1) Diagnosis of a schizophrenia spectrum disorder (schizophrenia, schizoaffective disorder, schizotypal or delusional disorder). 2) Under the care of secondary care (specialist) mental health services. 3) Self report of a sleep disturbance in terms of sleep timing, duration, initiation or maintenance, or a mixture of these issues. 4) Some degree of dissatisfaction with sleep. There was no specific exclusion of those who had received specialised psychological sleep treatment, but due to lack of availability it was anticipated that few would have received this. It was planned to exclude those whose sleep problems were predominantly respiratory (sleep disordered breathing), primarily a parasomnia (sleep paralysis, restless leg syndrome, periodic limb movement), and those with narcolepsy, severe learning disability, or moderate or severe dementia; in practice no participants with any of these exclusions requested to participate or were referred. Information on diagnoses and clinical problems was obtained from participants or gatekeepers, and checked against clinical notes with participant consent.

### Recruitment

Participants were recruited between January and May 2015, through posters, service user meetings, and via clinical staff. Recruitment through posters and user forums aimed to reduce the impact of gatekeeper filtering; staff are reportedly less likely to approach treatment non-adherent patients [34]. Equally those receiving less frequent input might not have been accessed through an approach relying solely on gatekeepers. A small payment for time was offered (£15 voucher). Participation was voluntary and only with informed consent. Due to the methods used it is not possible to comment on all those who may have been eligible, had information, and chosen not to participate. Of those referred and spoken to by the researcher (SF) two did not participate: one was discharged from the ward before the scheduled interview and contact was lost, another did not want to disclose their diagnosis or allow the researcher to contact their care team. No participants dropped out.

### Data collection

Interviews were conducted by the lead researcher (SF), who the time of data collection had prior experience of designing, conducting and publishing qualitative research. She was qualified to BSc (Hons) level, was experienced as a mental health Occupational Therapist, and was undertaking a Masters in Clinical Research. Participant information sheets included the interviewer’s profession and explained that the project was part of a Masters course. It is likely that participants assumed the interviewer had a personal or professional interest in sleep. The interviewer did not have a prior relationship with any participants recruited.

Interviews took place between February and May 2015, in participant’s homes and on Trust and University premises (participant’s choice), and lasted between 30 and 110 min. One interview was completed in two parts on consecutive days to accommodate the participant’s attention span. One participant was accompanied by their care co-ordinator, the others opted to be interviewed alone.

Transcripts were not sent for participant checking, but transcripts were checked against the audio, and the audio was used during analysis. Demographic and clinical information (age, gender, diagnosis, level of support, and current medications) was collected from case notes, gatekeepers, and participants, and two clinical measures were rated, as below:

### Global assessment of functioning (GAF)

To record symptoms and functioning, the Global Assessment of Functioning (GAF) was rated soon after participation, with the assistance of the care team where possible. The split GAF is found to be valid and reliable within this population, although admittedly best results are achieved with two raters [35]. The GAF is the most commonly used functioning scale in psychotic illnesses [36], is quick to rate, and no training is required. Guidance is available [37], which the researcher became familiar with.

The split version of the GAF was chosen, as advocated by various authors [38, 39], to preserve separate ratings for symptoms and functioning (the regular GAF records only the lowest of these values).

### The Pittsburgh sleep quality index (PSQI)

The PSQI [40] was selected as it is the most widely used self-report measure of sleep [41], allowing comparison to other study populations. The audio recorder was started before PSQI completion and participants were encouraged to discuss their answers, creating an easier opening to the interview, as participants tend to find questions about behaviours easier to answer than those about beliefs and feelings [42].

### Interview style

The semi-structured interview then commenced, either starting as per the question schedule (Additional file 1), or in response to participant’s comments during completion of the PSQI. During design service user feedback was obtained on the question schedule, this was also tested with a healthy volunteer. Topics were memorised to reduce the interviewer’s reliance on a prompt sheet and increase responsiveness to the participant [43].

The interviews explored experiences and beliefs regarding sleep, then moved on to elicit attitudes to interventions. Although not adhering rigidly to the schedule, it was important to try to keep these main topic areas roughly in the planned order, in order that more open questions preceded more specific questions about particular intervention approaches (‘funnelling’), so as to be less leading [44]. Follow-up questions were improvised to further explore topics participants raised. Detailed prompts were planned for participants who give short answers, yet remained happy to continue (Additional file 1).

Field notes, and reflective notes regarding interview technique, were written following interviews; these were added to during transcription and analysis, to support reflexivity and improve interviewer performance [45]. Reflections regarding the interviews were discussed within the team, after the first three interviews the most substantial revision to the question schedule was made through a discussion with PB; creating interventions cue-cards (Additional file 1).

### Patient and public involvement in design

As well as discussing the question schedule with service users, as above, consultation covered research aims, and the readability, presentation and content of recruitment materials. Service users were accessed via staff, and via user-groups, feedback was received in person, by phone or email. A small payment for involvement was given (£5 voucher).

### Analysis method

A phenomenological approach was deemed most relevant to the objectives and Interpretive Phenomenological Analysis (IPA) was selected. IPA fits with the researcher’s critical realist perspective; positing the reality of the outside world, but acknowledging the fallibility of our knowledge of this world [46]. IPA acknowledges that experience is subjective, but asserts that it is a meaningful exercise to attempt to understand the thoughts and experiences of others, even if this understanding may never be absolute [44].

IPA was chosen in favour of a descriptive phenomenology for its focus on the individual’s interpretation of experience, not just the nature of the experience (the so-called *double hermeneutic)* [47, 48]. There is also a precedent for the use of IPA to explore health-related beliefs informing future treatment [49]. Previous work has identified that sleep is something which people share experience of but which many rarely discuss in any detail, and about which people can carry many implicit assumptions [50].

**Fig. 1:**
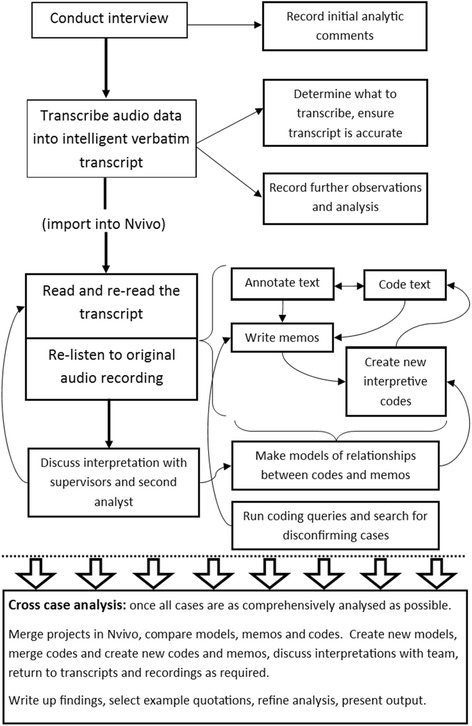
The analysis process

In IPA each transcript is analysed individually, attending first to each’s nuances and contradictions, and resisting immediate comparison to other cases [26] (Fig. 1). It was anticipated that experiences and perspectives of sleep would be highly personal, and diverse, thus the pooling of data from the outset was not desired, as it would result in a more superficial analysis.

Coding text is not an inherent feature of IPA, many authors suggest simply annotating text and writing memos [44]. The decision to use coding and to use Nvivo, was influenced by the larger than usual sample size for an IPA project, bringing increased need for systems for data management, retrieval, and auditability [51]. Some a priori codes were used to label and index the topic of the content (e.g. ‘snoring’), whilst other codes were interpretive and data driven (e.g. ‘searching for a cause’). Please see Additional file 2 for details of the method adopted to ensure a true case by case analysis whilst using Nvivo, and for details of the handling of a priori versus interpretive codes.

An active reflexive approach was taken, questioning personal perspectives and influences [52], assessing their validity against the data, and exposing them to scrutiny within the team [53]. This was achieved through discussion of interpretations with co-authors, and through independent analysis on selected transcripts by the researcher’s clinical mentor (see acknowledgements). Rather than seeking inter-rater reliability, or ‘confirmation’ of analysis (p87) [54], differences in analysis were often discussed to find that both perspectives contributed to a subtler, more coherent explanation [53], a more complete picture, and a more inclusive view [55]. Participants were not requested to comment on the findings, although they were each sent a study summary by their preferred method (email, post, via staff).

## Results

### Sample characteristics

Demographic and clinical characteristics of participants varied, and included participants at various stages of recovery from illness as shown by the GAF-F and GAF-S scores representing levels of functioning and symptoms. The sample was predominantly middle aged (Table 1), under-representing younger people with psychosis.

**Table 1:** Demographic and clinical information

		*n* = (of 15)
Psychiatric diagnosis:	Schizophrenia	8
	Schizoaffective disorder	6
	Delusional disorder	1
Current level of support:	Independent accommodation, outpatient care	2
	Independent accommodation, community team	8
	Independent accommodation, daily support	2
	Supported accommodation	1
	Acute mental health ward	2
Psychotropic medications prescribed:	Hypnotics	1
	Mood stabilisers	5
	Anti-depressants	9
	At least one antipsychotic	15
	One antipsychotic	8
	Two antipsychotics	7
	Oral antipsychotics, no depot^a^	11
	Depot and oral antipsychotics	3
	Depot antipsychotic, no oral antipsychotic	1
	Clozapine^b^	2
Antipsychotic dosage (as a fraction of DDD^c^)	Range	0.36–5.5
	Median / mean (SD^d^)	2 / 2.19 (1.28)
Gender:	% male	67%
Age:	Range	23–69 years
	Median / mean (SD)	44 / 45.9 (10.55)
GAF-F score (functioning)^e^:	Range	38–85
	Median / mean (SD)	65 / 62.3 (12.16)
GAF-S score (symptoms)^e^:	Range	21–80
	Median / mean (SD)	39/ 47.7 (19.40)

^a^Long-acting intramuscular injection, typically given every 1–4 weeks. ^b^Specified separately as only used in otherwise treatment resistant illness. ^c^The defined daily dose (DDD) is the assumed average maintenance dose per day for a drug used for its main indication in adults. [98] ^d^Standard Deviation. ^e^Scores may range from 1 to 100, lower scores = more impairment

Participants’ sleep complaints varied; in self-reported objective parameters such as sleep duration and sleep latency, and in what they considered to be problematic. Some participants also discussed having worse problems in the past (6/15). There were no criteria to exclude participants who had received targeted non-pharmacological sleep treatments such as CBT-i, but none had. This is similar to the low level of patients who had received CBT-i found in a larger secondary mental health survey sample [56], and perhaps reflects lack of availability.

PSQI scores ranged from 3 to 18 (mean 8.7). These are higher scores than reported in general population and non-clinical samples [57–59], suggesting more and worse sleep problems, as might be expected in this group. These scores should suggest a sample ranging from those with multiple and severe problems, to good sleepers, although it is noted that those obtaining relatively low scores often described severe problems in interview. PSQI scores and raw values are presented in Table 2.

**Table 2:** PSQI scores and individual PSQI question answers

Respondent	Self reported average sleep duration: hrs	Self reported sleep latency: minutes	During the past month, how often have you taken medicine to help you sleep (prescribed or “over the counter”)?^a^	Total PSQI score^b^
r01	11	60	Not during the past month (0)	3
r02	4.5	3	Three or more times a week (3)	12
r03	12	10	Three or more times a week (3)	6
r04	6	60	Not during the past month (0)	2
r05	14.5	180	Not during the past month (0)	8
r06	6.5	60	Not during the past month (0)	10
r07	4	120	Three or more times a week (3)	15
r08	8.5	20	Three or more times a week (3)	7
r09	11	30	Not during the past month (0)	7
r10	4.5	30	Three or more times a week (3)	18
r11	8	30	Not during the past month (0)	4
r12	3	180	Not during the past month (0)	13
r13	8	60	Three or more times a week (3)	9
r14	9	10	Not during the past month (0)	3
r15	7	60	Three or more times a week (3)	13
Median / mean	8 / 7.83	60 / 61		8 / 8.7

^a^Each question can score between 0 (best) and 3 (worst)

^b^Scores can range from = 0 (best possible), to = 21 (worst possible), although in practice scoring 3 in all areas would be very unusual even amongst those with severe sleep problems. Interpretation guidance suggests scores of >5 indicate poor sleep quality [40]

### Results

Analysis identified six themes:

- ‘Sleep priorities: sleep quality and sleep maintenance’
- ‘Loss of normality’
- ‘Knocking yourself out’
- ‘Priorities and life goals: daytime functioning’
- ‘Sleep as an escape: surviving’
- ‘Attitudes to non-pharmacological interventions: imperceptibility of gradual effects’.

Within each theme commonalities and contrasts, between and within accounts are described. Please see Additional file 3 for data excerpts, codes used, and their relationship with the themes and sub-themes.

### Sleep priorities: Sleep quality and sleep maintenance

Good quality sleep was seen as supportive of wellbeing, but was unfortunately for many, not a regular experience, and was characterised by some as a thing of the past. Good quality sleep was referred to as deep, proper, natural, or “like a child” (r09). Participants associated better quality sleep with falling asleep relaxed, with a sleep which was *needed* after a busy day, and with waking feeling refreshed. Understandably sleep itself was difficult to describe, due to its characteristic absence of consciousness, but participants expressed the belief that the nature and quality of sleep itself varied depending on a range of factors. Sleep maintenance was most valued; many cited getting continuous and unbroken sleep as the most important factor they would like to improve, and there was a consensus that interrupted sleep was less adequate:

“…it's rare that I have a, like, proper sleep, like it's a long time since the last time I slept right through the night without getting up.” *(r06).*

Many participants described deep sleep as perceptibly different, and superior, to shallow sleep:

“Sometimes you have a sleep, and you [still] feel tired, and you think... you feel like you can remember all that time. You know you got some connection to the time that you spent asleep, sometimes you’ve got no connection, it just feels (clicks fingers).” *(r13).*

Most participants were obtaining what is considered in general guidelines as sufficient sleep duration [60], but their perspectives on this varied widely. There were those who thought sleep is beneficial, therefore the more sleep the better. Some who thought this, slept for unusually long periods, whilst others felt they had to make a compromise to maximise their time awake. Others believed there was an optimal amount of sleep for them, too much being a waste - “not very productive” (r05), and furthermore - possibly bad for health.

Sleep latency (length of time to fall asleep) was more variably perceived. A large minority experienced even a relatively short sleep latency (e.g. 10 min) as abnormal and distressing. Those who described worrying at night, found the experience troubling and were keen to reduce sleep latency:

“…because, the voices, and there’s too many things on your mind and you get anxious and stuff like that but it’s...yeah, it is quite hard to get to sleep, stressful.” *(r15).*

These experiences are relatively typical of people with insomnia in isolation, and insomnia co-morbid with various conditions, who generally find prolonged sleep latency troubling, and over-estimate the length of time taken to fall asleep [61]. More surprisingly, many participants found long sleep latencies (e.g. 1 h or more) no problem, and some assumed this was “about average” (r01) amongst the population. It could be suggested that these participants had over-estimated their sleep latency, however studies have shown sleep latency was more accurately estimated by people with schizophrenia than healthy controls [62], these authors suggest a lack of over-estimation is due to a lack of negative emotional reaction to the failure to fall asleep. This relates well to the accounts of those participants who took a long time to fall asleep, but for whom this did not seem to be driven by anxiety, and who were not particularly bothered by this.

Daytime functioning was a shared priority, however a variety of often complex perspectives were expressed regarding sleep’s impact on various aspects of daytime functioning. This forms the focus of the theme ‘Priorities and life goals: daytime functioning’.

### Loss of normality

Many contextualised their sleep in a narrative that included significant loss associated with their illness; both tangible losses such as jobs or friends, and less tangible loss such as feeling “not normal anymore” (r02), or ambitions which now seemed unattainable. Loss in relation to sleep was expressed clearly and explicitly by some, and was present more subtly in other accounts, appearing to underlie the minimisation and re-framing of sleep problems described below.

Varying degrees of hope, acceptance, or resignation were expressed, from feeling problems with sleep were permanent, like psychiatric diagnosis, to feeling empowered to make changes, with a tentative expectation of success:

“…I know I'm going to be like this forever, there’s no point in telling a lie to myself, I know I'll always have problems sleeping.” *(r02).*

“I think, if I'm determined to make it improve, I think it would.” *(r03).*

Some expressed contradictory positions within their account, suggesting internal conflict, and incomplete acceptance:

“No I’d just used to sleep straight through. […] It was better. I think the best bit is that. You know, yea I do have memories of like when I was a kid and all that. Beautiful sleep. You know. But, erm. I’m not bothered.” *(r13).*

It was noted that those who were resigned or accepting of sleep problems employed passive coping strategies, used humour, or minimised or re-framed problems:

“I try and stay optimistic, you know what I mean, you know I wouldn’t call it a bad night’s sleep, I just say, you know, ‘a bit of overtime’.” *(r13).*

Most readily connected their sleep to their mental state, attributing stress or anxiety as a cause of poor sleep. They saw psychiatric conditions as affecting sleep, generally speaking, and for themselves. Many cited lack of sleep as a warning sign or relapse trigger. Although some expressed with resignation that their sleep was connected to their mental health, others felt more empowered and hopeful. Some described an adaptive acceptance of change; for instance describing taking extra care to look after sleep because of their condition:

“…in terms of looking after myself […] I can’t be staying up all night because if I do that for two, three, four nights, five nights […] it’s just I rather like that I’m symptom free and I do not want to jeopardise that. So in terms of sleeping I have to have routine. I’ve realised that now.” *(r04).*

In a further expression of perceived different-ness, many participants saw general-population guidelines as inapplicable to those with psychotic illnesses. For instance whilst some quoted commonly recommended average sleep durations, others stated they required more sleep because of their illness or their treatment. It is not known whether longer sleep than average might be required by some people with schizophrenia spectrum disorders; it is feasible that this may be needed to compensate for alterations to sleep architecture [22, 63], however this could equally be an understandable but incorrect extension of associating extreme sleep loss with psychotic episodes.

Most were aware of advice regarding keeping regular sleep times, but many advocated that, “it depends on the person’s make-up” (r02), and described napping or catching up on sleep as good strategies for those with mental illnesses in maintaining wellness. Many participants held assumptions of separate illness defined norms for sleep. Distinct norms may explain why so often when asked about ‘good sleep’ participants were initially unsure how to answer and then implicitly or explicitly distinguished between good sleep - in the general population, and good sleep - for themselves:

“Umm, not very good […] Not like other people…go on like sleep’s supposed to be, you know? […] Yes, I’ve had a few good night’s sleep but I still wake up early in the morning.” *(r12).*

Even those who’s sleep had improved much over the years, questioned the quality of their sleep compared to that of someone medication-free without a diagnosis, often feeling it was, or must be, inferior:

“I think perhaps I have very good sleep now, but the sleep I have is not natural sleep, it can't be, but I feel fine when I wake up in the morning.” *(r08).*

Illness specific definitions and expectations about sleep were therefore common between participants with differing sleep experiences and differing outlooks. These included lowering of expectations resulting from poor self-efficacy and self-stigma, and adaptive recalibrations to accommodate altered sleep needs.

### Knocking yourself out

This theme describes forceful methods of inducing sleep, their perceived drawbacks and consequences, and the circumstances seen as justifying their use. Firstly hypnotics; these were seen as knocking yourself out and were viewed negatively. Participants felt they were effective, but that overall their drawbacks outweighed their benefits, these included side effects, drug interactions, tolerance or addiction, unrefreshing sleep, or unnatural (‘false’) sleep:

“They say about sleeping tablets, but I wouldn’t want them, because you rely on them then to get sleep, and that’s a false sleep then isn’t it?” *(r10).*

Many participants’ current sleep problems did not merit hypnotics (e.g. oversleeping). Some spoke hypothetically, others had past experience, from during a psychotic relapse. These participants gave mixed reports, some felt hypnotics had been effective and necessary, others had found them ineffective, or complained of side effects. Even when advocated, hypnotics were described as a last resort:

“…it’s one of my last defences, like. These voices like to see me awake and agitated…” *(r13).*

All except one participant expressed nuanced or conflicting views regarding antipsychotics (one participant felt medication was unnecessary). Concerns about antipsychotics or other psychotropic medication in relation to sleep closely paralleled those expressed regarding hypnotics, this participant states the similarity:

“…all the sleeping pills that have been around are addictive […] what I take, is not known to be addictive but it's… I would think the brain becomes used to having certain chemicals, on a regular basis, and that way you can't do without them so it's almost like they are addictive, but not in the sense that sleeping tablets are.” *(r08).*

Like a hypnotic, but seen as more acceptable, most felt antipsychotics helped with their sleep, and some were very thankful for this. A recent review confirms improvement in some sleep variables by clozapine, olanzapine and paliperidone (whilst quetiapine further disrupted sleep) [64], however antipsychotics have not been shown to improve sleep variables to normal levels, and authors describe anti-psychotics as an inadequate treatment for the sleep problems present in most people with schizophrenia [65, 66]. For many participants, antipsychotics were viewed as a form of sleep treatment, highlighting that although professionals may mostly view effects on sleep as side effects [67], many participants viewed them as intended effects:

“I take it [oral anti-psychotic] at night, because it’s like you’re sleeping tablets sort of thing as well…” *(r10).*

Antipsychotics being viewed as a means of inducing sleep was also reflected in PSQI answers; only one participant was prescribed a hypnotic, yet seven participants answered ‘every day’ regarding question 7 on ‘medicine to help you sleep’ (p210) [40] (Tables 1 and 2).

Neither hypnotics nor antipsychotics were deemed ideal, but whilst hypnotics were a last resort, antipsychotics were a compromise. Participants viewed hypnotics as appropriate only for short term use, and antipsychotics as more acceptable for long term use, reflecting prescribing guidance [13, 68], perhaps echoing advice participants had received from professionals.

Medication’s effect on sleep and wakefulness was seen as negative if it caused over-sedation or excessive sleep. A few reported this as a major current problem, while for most this had been a problem at some time on different medication or dosage, but was now a minor and tolerable side-effect. For less obviously identifiable reasons, participants also suspected or assumed that sleep-inducing medication might alter sleep, making it inferior to natural sleep. Some expressed that sleep without medication (whether feasible for them or not), would be qualitatively different, and would be more beneficial:

“I'd actually sleep, yeah. And I think I'd be able to do more things as well, you know, in the day, if I wasn't on the medication, sometimes, if I managed to get natural sleep.” *(r03).*

Systematic reviews of laboratory sleep studies, indeed show support for the view of antipsychotics as improving some parameters whilst worsening others [69, 70].

Perceived reliance on medication was a compromise, as it did not promote a sense of control, but remained preferable to not sleeping:

“I know I just wouldn't sleep so, I rely on what I take. But on the one hand I don't like the idea of being controlled by medication, but if that's what I have to do then that's what I have to do to stay okay.” *(r08).*

Sleep which was unpredictable, was more distressing, and meant more forceful measures to gain control of sleep may be deemed necessary; forceful measures included alcohol or illicit drugs to induce sleep, or purposely avoiding trying to sleep until exhausted, to then fall asleep quickly. This participant usually slept in the lounge with the TV on, except for when they had purposely stayed up all night to fall asleep more easily the next day:

“I sleep, if I'm really, really, tired I can sleep well in [my bedroom], but I find it difficult to fall asleep in there.” *(r14).*

Sleep disturbed apparently without cause, could even be experienced as interfered with by external forces. By contrast more knowledge and understanding of sleep often meant sleep was seen as being affected by *known causes*, which could voluntarily be altered, resulting in a greater sense of control, and less frustration and desperation for forceful measures:

“Yeah, because sometimes I'm trying to go to sleep, and I just, I'll hear a subject pop-up, and then...I can feel myself coming back around again. You know I'm like, pfff, that's flipping, nothing to do with me, know what I mean, and it's just positive energy.” *(r13).*

*“…if I get up really late then it affects the time I get to sleep the night after you see.” (r04).*

Knocking yourself out, in its various forms, was a potentially necessary measure to take control of sleep, but those who felt they had gained some control of their sleep through less forceful means, expressed more satisfaction.

### Priorities and life goals: Daytime functioning

Participants sometimes digressed from matters relating to sleep to more personally important topics, and sometimes explicitly stated that sleep was low priority:

“I don’t really care about my sleep, as long as I’m not sleeping in the hospital.” *(r12).*

Sometimes it was not considered an option to prioritise improving sleep problems, because (as above) they had been accepted:

“I'm used to it now so is neither here nor there, it's secondary...it's like I've been through the first phase of realising that my sleep is not normal anymore…” *(r02).*

Sleep was generally not a priority in itself, but participants described many links between sleep and their priorities, such as their life goals (personal, vocational, moral and social). Some explicitly described sleep impacting on their functioning, whilst others were more conflicted, and inclined when questioned to deny that poor sleep had any effect on their daytime life, but then go on to describe various impacts they did attribute to poor sleep. Functional benefits attributed to adequate sleep included feeling more sociable, increased energy and motivation, thinking more clearly, and differences in waking experience which were tangible but difficult to quantify. This participant describes how if you don’t get sleep you feel “a bit funny” (r13), whilst after good sleep:

“It just feels, it feels good. I feel I can connect to my nerves a bit better, it feels more, more, more sensitive […] them senses get ignored quite often, so it’s not really a big issue really, but I do notice it, you know what I mean, I say to God ‘yeah, this is how I should be’.” *(r13).*

Sleep timing and duration were both seen as important for participation in time-dependent social activities, and quality of life. As described earlier, some perceived a need for longer than average sleep to support functioning and wellness, but wanted a shorter sleep duration to leave more time for valued activities:

“I don’t want nine hours’ sleep. Your life’s boring enough without sleeping too much.” *(r04).*

Participants described work and occupational goals, acknowledging that many of these were contingent on sleep, requiring an adequate amount and quality of sleep. Those with goals of paid employment saw it as particularly important that sleep was not over-long, and was predictably timed:

“I’d just like to go to bed at ten o’clock, sleep eight hours, wake up in the morning and go and do a job.” *(r09).*

This participant took a similar yet inverted perspective, reflecting that it was positive that they did not work, as their sleep could be unpredictable:

“Well, I don't work because basically I'm mentally ill [...] if I have a rough night and I don't sleep 'til about six, then I know that, you know, it's okay really because I'm not working, I've got no responsibility.” *(r11).*

One significant factor underlying the desire to better control one’s own sleep, seemed to be a requirement participants felt, to present themselves socially as in control of themselves, and therefore as alert. This was evident for instance where participants emphasised to the interviewer their alertness and ability to stay awake:

“I’m yawning now, but I don’t know why I’m yawning and stuff like that. I’m wide awake.” *(r09).*

Some participants stated this social requirement more explicitly:

“…in terms of socialising, and I guess you know, I’m a single bloke at the moment as well, but it’s just - since I’ve been on this treatment I don’t want to be falling asleep at midnight and, ‘what you falling asleep for?’” *(r04).*

As well as appearing in-control, participants wanted to avoid being considered “lazy” (r01). Thus managing sleep had implications for presenting a socially acceptable image to others, and for self-image. To avoid negative self-attributions some distinguished between how their behaviour may appear, and their nature:

“I’m sure that a lot of people would say, it’s just plain laziness. But if it was just plain laziness, I wouldn’t feel guilty.” *(r05).*

For daytime-life, alertness was important, and increased sleep inertia, or not feeling fully awake, were frequently emphasised complaints. Improving “the recovery after sleep […] coming too quicker” (r02) was desired by many. Increased sleep inertia was attributed by many but not all, to side effects of psychotropic medications. Sleep inertia was not always proportionate to the recentness of taking medications however, as one might expect for a hangover effect. This participant suspects their medication increases sleep inertia, and yet:

“[if I have a lie in] it’s then that I get so deep…whether it’s deep sleep I’m not too sure, but I find it easier to get up at eight than I do at ten.” *(r04).*

Although deep sleep was seen as beneficial, many found it difficult to wake from. This presented a dilemma for some, as the sleep they described as ‘good quality’ did not necessarily result in better daytime functioning in the short term. This participant would avoid deep sleep when they had morning commitments, by sleeping with the bedroom lights on, so as to wake on time:

“It just means that I sleep lighter, and I do sleep, but perhaps toss and turn a wee bit.” *(r05).*

Also for some, waking was such a difficulty that if an appointment was too early, their strategy was to stay up all night to reliably attend, then sleep after the appointment.

### Sleep as an escape: Surviving

There was a significant exception to the rule that sleep was seen as a means to an end to support daytime-life. This was where sleep, and dreams, were occasionally valued in themselves for pleasure:

“It’s just so relaxing. I enjoy going to sleep now […] But my dreams now are just so relaxed where I don’t want to get out of bed because it might ruin the dream.” *(r09).*

Sleep could also be used as an escape; from symptoms, from thoughts, or from reality. As opposed to *pursuing life goals*, some accounts emphasised *surviving*. When “it's not really a nice world, to be awake in” (r13), then sleep and dreams, were welcome respite:

“It's being away from all my problems, it's heavenly […] leaving this area behind and just waking up somewhere else like 150 years later, that makes me happy.” *(r02).*

Reliance on sleep as a coping mechanism increased the feeling of a lack of control when sleep was broken or not deep - providing an incomplete escape, or was interrupted by distressing nightmares, which for some was a frequent occurrence.

### Attitudes to non-pharmacological interventions: Imperceptibility of gradual effects

For tabulated opinions regarding specific interventions see Additional file 4, this theme describes the patterns, contrasts, and rationalisations identified within intervention opinions. Opinions varied, and some participants were more positive than others about sleep interventions in general, and more hopeful regarding improvement in sleep.

Past experience of sleep interventions varied, a few said sleep was never discussed. No participants reported being referred for targeted non-pharmacological sleep treatment (except one who was seen by a sleep specialist regarding sleep apnoea). One described receiving a behavioural relaxation-based intervention from their occupational therapist to target sleep and anxiety. Participants very rarely complained that they should have had more help than they had received.

Participants had often received sleep self-help advice from their mental health team, although they did not always identify this straight away, some initially assuming that sleep advice would come from elsewhere, or from a separate specialist:

*“Well as long as the nurses and the doctors recommend someone to see you then they’ve obviously got a qualified professional opinion and that’s good enough for me.” (r15).*

Participants described sleep problems’ as highly individual and context dependent, and interrelated with multiple factors which were hard to unpick. Analysis revealed an unquestioned assumption that sleep problems were complex and time consuming to assess or address:

“…maybe not GPs because I don't think they would have much time to sit with you and try to find out what would be causing your inability to sleep.” *(r08).*

Using self-help approaches for sleep was described as part of a gradual process of recovery, learning and self-discovery:

“With the benefit of hindsight I’ve learned so much over the years […] I learn something new about myself every day…” *(r04).*

This learning for many included gaining knowledge of sleep hygiene principles and self-help approaches, and experimenting to determine their individual appropriateness:

“[Mindfulness] So that definitely works for some people, but not… it didn't work for me. […] Yeah I stuck out the whole course, it didn't do any harm but I don't think it...it hasn't been a life changer! (laughs)” *(r08).*

Trial and error was used to find effective ways of relaxing, and some experimented with adjusting bedtime and daytime routines. Many experimented with medication timings, which they felt was a helpful process, whilst others stuck exactly to prescriber advice.

When participants advocated self-help measures this was rarely a single measure as an instant solution; successful approaches were integrated into the whole lifestyle. This was particularly apparent in relation to routine based approaches. Strong feelings were expressed regarding routine; many who were in a regular routine, or who had been in the past, felt routine was really essential. Many more recovered participants made very significant efforts to maintain regularity in their sleep routine. Their efforts are reminiscent of those described by patients with bipolar in qualitative [71] and quantitative studies [72], where participants described themselves as over-sensitive to small departures from sleep routine.

Other participants were more dubious of the contribution of regularity toward good sleep, and highlighted significant barriers, these often related to self-discipline, and a lack of initial reward:

“I don’t know how you can be busier in the day though, if you haven’t had a good night’s sleep.” *(r10).*

Many felt they may need support to adequately attempt strategies involving multiple and substantial behavioural changes. Occupational roles and external responsibilities were also identified as potentially supportive of regular sleep routine:

“…I think that would come if I got a job or I started work or started doing more.” *(r14).*

Some advocated certain ideas based on what they had read or been advised, however participants expressed stronger opinions regarding approaches they could relate to their experiences. The description of sleep restriction, for instance, often provoked positive reactions. It required little explanation as participants understood tiredness and sleep pressure and could relate to the mechanism of its action:

“Yeah. I think reduce hours in bed at night. So […] your body's going to be tired throughout the day. So I think that would help.” *(r11).*

It is known within sleep research, that regular exposure to environmental cues, especially daylight, regulates circadian rhythm [73, 74]. Understandably, participants were unaware of the gradual entraining effect of light on circadian rhythm, as no doubt are many of the general population. Some participants reported keeping their curtains closed during the day due to paranoia, or a simple lack of net curtains; without an understanding of circadian rhythm they were unaware of the gradual impact of this action upon their wellbeing:

“Yeah, yeah. It [being dark indoors] doesn’t make me any more tired or make me want to go to sleep...” *(r10).*

Participants largely agreed with recommendations around relaxing bedtime routines, having experienced the acute alerting or calming effect of light levels and other environmental stimuli. Notably many reported continuing to follow these practices even though this had not solved their problems; the effect was perhaps perceptible even if it was insufficient. By contrast approaches such as reducing napping, and increasing daytime activity and regularity, which have a gradual or delayed effect on the ability to sleep, received more varied evaluations. Sometimes approaches were dismissed, after participants tried them briefly or inconsistently, and experienced no improvement. By contrast to *knocking yourself out* – which was risky but effective, more subtle approaches were sometimes seen as safe but ineffective.

## Discussion

This is the first study to explore the experiences and priorities of people with established schizophrenia spectrum disorders regarding sleep and sleep treatment, outside of the context of an experimental study. Participants situated changes to sleep alongside changes to life and self, associated with the diagnosis of a psychotic illness. Sleep since illness was typically perceived as fundamentally altered, and sometimes as diverging from social norms. Changes to sleep included in respect of timing, maintenance, refreshing-ness, quality, and perceived reliance on medication for sleep. Personal responses to perceived changes varied and were influenced by individual sleep expectations, hope for improvement, and sense of control.

Hypnotics and antipsychotics were discussed amongst forceful measures to control sleep. Neither were viewed as ideal, but antipsychotics were viewed as a more acceptable compromise. Less forceful approaches to improve sleep gained very mixed reviews, their value was described as heavily context dependent, and they engendered more subtle effects which were harder to verify. Sleep was mostly viewed as a *means to an end*, to support waking life. However if waking-life became distressing the meaning of sleep could alter, and sleep could become *an escape*.

The use of sleep as an escape has also been described in practice observations of staff delivering CBT-i in people with hallucinations and delusions [66], and by participants with major depression with suicidal thoughts and behaviours [75]. Similarly some of these participants described preferring sleep to waking life, and using sleep as a coping strategy to escape suffering (although their distress was due to negative thoughts rather than hearing voices). Similarly also, many simultaneously worried about daytime sleep indicating laziness, and being “a waste of life” (p5) [75].

Daytime functioning was seen as important, and as being supported by good sleep quality, however these constructs are relatively poorly represented in existing self-reported sleep measures. A review notes that existing measures tend to neglect these focusing instead on the more easily quantifiable factors of sleep latency, sleep duration and sleep maintenance [76]. Many measures also do not ask regarding satisfactory timing of sleep [40, 41], or sleep inertia [40, 41, 77], which in this population had a significant impact on social and occupational daytime functioning.

Among study participants undisrupted sleep was seen as better quality, which aligns with research in healthy subjects [78, 79]. ‘Broken’ sleep was a common complaint, which participants felt impacted on them, and although objective sleep measurements were not taken in this study, subjective and objective sleep continuity and sleep quality have elsewhere been found to predict next day functioning and symptoms in this population [18]. Participants may also perceive more awakening or incomplete sleep due to increased eye-movement density during Rapid Eye Movement (REM) (indicating physiological arousal), and increased light sleep (stage 1) [22].

An unspecified sense of *different* sleep, or sleep which was *not proper sleep*, since illness has been described, including by those who self-report screening tools may not identify as having a sleep problem. It should be acknowledged that this could be purely due to perception of oneself as *different*, however this did not ring true for all accounts. There are a number of potential biological contenders to explain a qualitative difference in sleep. Evidence shows a reduction in stages 3 and 4 of sleep, and in slow wave sleep (deep sleep), and an increase in stage 1 sleep (light sleep) in schizophrenia as compare to healthy controls. In earlier stages of illness evidence shows altered timing of the first period of REM, although this may be due to medication. Alterations to the microarchitecture of sleep (including sleep spindles) may also play a role, but findings regarding these are as yet inconclusive [22].

Antipsychotics and other psychotropic medications were prominent in participant’s accounts regarding sleep, so it is interesting to note their low profile in existing qualitative literature regarding sleep experiences in people with serious mental illnesses [23]. Views regarding hypnotics have by contrast been well explored within previous studies, and agree with those of the current study [23].

For most, some effect on sleep was attributed to illness. It is significant that even those with less insight into other symptoms connected sleep disturbance with their diagnosis, perhaps as acute sleep problems when unwell can be more concrete, and easier to appreciate than symptoms such as unusual thinking patterns. This agrees with the suggestion that awareness of non-core symptoms including mood and sleep symptoms may account for the substantial proportion of patients who believe medication is necessary despite lacking awareness of the core-symptoms of psychosis [80]. Although improved compliance is perhaps welcome, through this connection many participants saw sleep disturbance as a permanent accompaniment of their diagnosis; when actually in many cases there is reason to believe sleep can be improved. Professionals may have unwittingly contributed to this connection by re-enforcing early signs of insight when patients often first connect mood or sleep symptoms to a possible illness.

Adding to this the view of antipsychotics as a targeted treatment for sleep, or exaggerated perception of their effectiveness for inducing sleep, may add to a perception that patients’ sleep problems are beyond help; as they believe they are taking a treatment which has not worked. This may also be disempowering as where people overly credit their antipsychotic with improving sleep, they may fail to credit behavioural or psychological changes, in which they played a more active role, which helped improve sleep.

The acceptance and low prioritisation of sleep problems contrasts to findings in samples with insomnia without psychiatric co-morbidity, these samples described sleep problems as more significant than other people recognised, and deserving more attention [81–83]. By contrast many participants in the present study had very poor sleep as defined by PSQI scores, or self-reported quantitative parameters, reported minimal input regarding this, and were accepting of a lack of any improvement. This is despite sharing the priorities of those with insomnia without co-morbidity in respect of: desiring sleep maintenance, valuing daytime functioning, and experiencing social pressure to appear alert [81–83].

In models of chronic primary insomnia worry about sleep, and hyper-arousal play central roles in the maintenance of insomnia [84], insomnia treatments therefore often focus on reducing sleep related worry. Participants in the current study have described many experiences of sleep disturbance where worry plays a lesser or different role. A long sleep latency can also be caused by insufficient sleep-pressure due to too recent or too much sleep [1], by circadian dysregulation [85], or by reduced daytime activity [86]. The present study’s findings agree with practice observations which emphasise the importance of addressing these other factors in this population, and describe worry about sleep as being a less essential treatment target [66].

Consideration of other aspects of functioning and life quality, beyond and apart from symptoms of mental illness, is essential to recovery orientated practice in mental health [87]. The personal priorities which participants have highlighted as being affected by sleep link closely to aspects of recovery identified by service users in other research: identity, hope and optimism, control / empowerment, and contribution to society / connectedness [88, 89].

The connection between sleep and personal priorities was often linked to work. As well as regular sleep supporting work-readiness, participants identified the potential of regular commitments to bring about improvement in sleep patterns. Other research confirms service users found work stabilising, helping them cope with symptoms [90]. In longitudinal analysis work is linked to improvements in wellbeing, functioning and symptoms [91].

It is known that interventions which regulate circadian rhythms can improve the course of illness in bipolar [92], and similarly many more recovered participants in the current study felt routine based approaches to improving sleep had played a crucial role within their recovery. Circadian dysregulation is thought to cause some of the cognitive impairment associated with schizophrenia [19], while a more regular rest-activity cycle is associated with better frontal lobe performance [17], and more adaptive coping styles [14]. Both previous research, and participants accounts, support the notion of a synergistic relationship between circadian regularity and occupational functioning in schizophrenia spectrum disorders; with regular valued activities giving exposure to environmental and behavioural zeitgebers, improving circadian rhythm, thereby improving functioning and symptoms, thus promoting further improvement in the ability and motivation to engage in valued activities.

### Implications

Any recovery orientated assessment should evaluate the impact of sleep on the person’s ability to engage in the social and occupational roles they prioritise. Limited patient motivation toward sleep [93] may be due to believing change is not possible, rather than feeling the matter is unimportant. Patients may not bring up sleep problems or expect help, reaffirming recommendations that assessment should be consistent, direct and proactive [50]. Global recovery focused Patient Reported Outcome Measures (PROMs) should include enquiry regarding the impact of sleep, as should any holistic assessment by an occupational therapist or another profession. To avoid sleep problems being overlooked it would also be beneficial if updates to policy and guidance regarding psychotic illnesses [12, 13] made explicit reference to sleep.

PROMs and other assessments of sleep should better capture the concerns of this population, especially regarding sleep quality and sleep timing. Findings concur with existing recommendations that sleep’s impact on daytime functioning requires multidimensional assessment [41], and the present study suggests sleep inertia and sleep timing should be part of this. Items should be developed with reference to experiences and priorities, and content validity and responsiveness should be tested within this population [94].

The question of how much sleep is required is pertinent, because always feeling you are getting insufficient sleep would cause undue stress, and because of the evidence that excessive sleep carries health risks [95]. Evidence from experience sampling suggests sleep duration is not the most important factor, at least in terms of the immediate effect on next day functioning; sleep continuity and sleep quality were much more predictive of functioning and symptoms [18]. In order to provide better population specific guidance the effects of differing habitual sleep durations in people with psychotic illnesses should also be investigated. This may be investigated safely and ethically through observational designs, and through longitudinal studies making smaller modifications to sleep duration, perhaps utilising experience sampling as above.

This population’s experiences may be dissimilar in certain respects to those of people with insomnia without comorbidity. It may take patients increased efforts to regulate their sleep timing, even when their symptoms are stable, and the causation and maintenance of insomnia may be less driven by anxiety regarding sleep. Psychotic symptoms and other sources of mental distress are likely to contribute to acute exacerbations of sleep problems, and to unhelpful sleep behaviours including daytime sleeping or avoidance of sleep. Further investigation of mechanisms would assist in the adaptation of models and development of interventions. Based on the similarity with experiences in bipolar, it is also recommended that approaches from bipolar [96] should be applied and adapted in this population. The present study’s findings suggest intervention development may fruitfully focus on approaches to regulating sleep routines through the timing of valued activities, to create a personalised, whole lifestyle approach. The mixed views regarding routine, highlight that methods for making gradual progress more easily perceptible to patients would be important, as would supporting motivation toward behaviour changes, and taking account of the actual and perceived effects of regular medications.

Prescribing decisions and discussions should take account of the actual and perceived impact of medication on sleep, and advice around sleep promoting effects of medication should be evaluated. This should not be overstated and the influence of other non-pharmacological factors should be included and emphasised. Because of the perceived significance of the effect of anti-psychotics on sleep, future studies of the effects and side effects of antipsychotics should include measures which better capture patient priorities regarding sleep and alertness [69].

### Strengths and limitations

It was possible to compare analysis with a second researcher for selected transcripts, however it is acknowledged that all data collection and most data analysis was completed by a single researcher, which may be viewed as a weakness.

Despite efforts to recruit widely, an element of volunteer sampling inevitably remained [29]; those approached by gatekeepers voluntarily self-selected to participate. Participant information specified ‘psychotic illness’, whilst some reject this label. Some researchers have found those whose recovery style was to integrate psychotic experience, were more likely to participate in qualitative research [34], compared to those who sealed-over and distanced themselves from these experiences [97]. Although people with varied insight participated there may be subgroups whose views are not well explored here; utilising a measure of insight might have been informative.

Participants were not screened for severity of sleep disturbance using a standardised tool. However, it was considered desirable to include the views of all those who viewed themselves as having a sleep problem, irrespective of their scoring on any predetermined measures. The contribution of participants who previously had more severe sleep problems also brought an unexpected strength to analysis, as triangulation was possible where some participants described current experiences, and others described similar experiences looking back with hindsight. This also meant that 15 participants, provided accounts of more than 15 relationships with sleep.

The breadth and diversity of experiences can be viewed as a strength or as a weakness. It is acknowledged that saturation may not be complete in all areas [28]. Sometimes analysis raised a question, but insufficient further data followed to answer this confidently. For example, whilst sleep in the context of oral antipsychotic use was well explored, it was not possible to adequately examine the contrast with the experiences of those on a depot injection only whose medication levels are more stable throughout the day.

## Conclusion

To enhance successful recovery for those with schizophrenia spectrum disorders, sleep problems require independent attention. Many theories and therapies developed in other populations can no doubt be effectively adapted for this population, but in order to optimise treatment there are important differences to take account of. One such difference, is the physiological and psychological effect of taking a sedating drug in the evening, with no plan to discontinue. The significance of this for a person should not be overlooked. Sleep experiences and priorities in people with schizophrenia spectrum disorders are of course affected by symptoms and diagnosis. However more subtle differences in the neurobiological and psychosocial mechanisms causing and maintaining sleep disturbance in this population are also important. Through further objective investigation, and exploration of experiences, existing models and interventions may be further adjusted and adapted.

## Additional files

Additional file 1: The evolving question schedule (original version and final version). (DOC 50 kb)

Additional file 2: Creation of separate Nvivo projects for each case. (DOC 457 kb)

Additional file 3: Additional data excerpts – themes, sub-themes and codes. (DOC 66 kb)

Additional file 4: Participant opinions on specific interventions. (DOC 106 kb)

## Abbreviations

CBT-i: Cognitive Behavioural Therapy for insomnia
GAF: Global Assessment of Functioning
IPA: Interpretive Phenomenological Analysis
PROM: Patient Reported Outcome Measure
PSQI: Pittsburgh Sleep Quality Index
REM: Rapid Eye Movement
SD: Standard Deviation
