# Supplementary material for: Experiences, perspectives and priorities of people with schizophrenia spectrum disorders regarding sleep disturbance and its treatment: a qualitative study
Source: BMC Psychiatry. 2017 May 2;17:158. doi: 10.1186/s12888-017-1329-8 (PMC5414297; doi:10.1186/s12888-017-1329-8)
Supplement: Supplementary file 1 — The evolving question schedule (original version and final version). (DOC 50 kb) [file 12888_2017_1329_MOESM1_ESM.doc]

**ORIGINAL VERSION**

- How would you describe your sleep?

*What is that like for you?*

*Would you say that you have a ‘sleep problem’?*

- What would you say really good sleep is like?
  - Can you tell me about a time when you had a really good night’s sleep?

*What was it like falling asleep? Being asleep? …and when you woke up?*

*What was it about that night’s sleep that makes it ‘good sleep’?*

*Why do you think you had such good sleep on that occasion?*

- Can you tell me about a time when you had a poor night’s sleep?

*What was it like getting to sleep then? Being asleep? …and waking up?*

*What was it about this that made this a poor night’s sleep?*

*Did any factors impact negatively on your sleep that night?*

- What positively or negatively affects how well you sleep?

*Anything about what you’ve been doing that day? / feeling? / the environment?*

*What affects people’s sleep? What have you heard affects others? (and you?)*

- How important is it to you to get good sleep?

*Compared to some other things, how important is sleep? (examples?)*

- Would you say that anything is affected by your sleep?

*Anything about how you feel / behave / manage / roles and tasks? …affected much?*

- Is there anything you have tried which helped your sleep? *(how did it help?)*
  - Is there anything anyone else has suggested, or done, which helped (friend, family, healthcare professional, book, internet or TV)? *(how did it help?)*
- Is there anything you have tried / anyone else has tried, that didn’t help?

*Why do you think that wasn’t helpful for you?*

- How likely would you be to seek treatment to improve any issues you have described now?
  - What would you most like to change?
- Which issues out of those you rated on the PSQI would you want to change?
- To what extent do you feel your answers on the PSQI showed your problems as you see them?

*What is shown well? What is not shown / not shown accurately?*

*How did you find completing the PSQI?*

- How do you feel about being given **information or advice**?

*Would you want information and advice on any particular topics?*

*How would you like to be given information and advice? (format, when, by whom?)*

- Would you want to follow **a structured programme** and complete **‘homework’ exercises** to help improve your sleep? *(tried before?)*
- Do you think that any **‘talking therapies’** would be helpful to improve your sleep? *(tried before?) How do you feel about receiving advice, therapies or interventions* ***online*** *or by* ***phone****?*
- How would you say you feel toward **relaxation, mindfulness or meditation**? *(tried before?)*
- How would you feel about making changes to:

…your **physical environment** (including lighting, opening curtains)?

*Tried before?*

*When?*

*What did you do?*

*Effective?*

…to **when** you do different activities in your day?

…the activities you do **in the hour before going to bed** (e.g. no TV/internet)?

…to what **activities you do during the day?**  Including **physical activity**?

…to your use of **caffeine, nicotine** (and other **drugs**)?

…to your **diet**? (timing, amount and type of food)

- How do you feel about setting certain **rules about what time** you go to bed and get up in the morning in order to get into a regular schedule? *(tried before?)*
- How would you feel about **restricting** the amount of time you spend in bed so that you’re more tired when you go to bed? *(tried before?)*
- What do you think the role of prescribed **drugs/medications** should be in this? *(tried before?)*
- Is there any **other** type of approach I haven’t mentioned that you think should be included?
- Out of all these approaches which ones do you think would be most likely to work?

*For you? For others? And why? (refer to list)*

- They mostly involve a large effort on your part. Which ones do you feel confident or less confident in being able to use well? *(refer to list)*
- How likely do you think it is that your sleep will improve in future?

**(END)**

**FINAL VERSION**

- How would you describe your sleep?

*What is that like for you?*

*Would you say that you have a ‘sleep problem’?*

- Can you tell me what it’s like when you have a poor night’s sleep?

*What’s it like getting to sleep? Being asleep? …and waking up?*

*What is it that makes this poor sleep?*

*Is there anything that impacts negatively on your sleep?*

- What would you say really good sleep is like?

Can you tell me what it’s like when you have a really good night’s sleep?

*What was it like falling asleep? Being asleep? …and when you woke up?*

*What was it about that night’s sleep that makes it ‘good sleep’?*

*Is there anything that contributes to you having good sleep?*

About the day before? About your bedtime routine? About how you’re feeling?

What do you think generally affects people’s sleep? Does that affect you?

- How important is it to you to get good sleep?

*Compared to some other things, how important is sleep? (examples?)*

- Would you say that anything in your life is affected by your sleep?

*Anything about how you feel / act / roles + tasks / physical or mental health?*

*Can you tell me about a time when something was affected by your sleep?*

- How likely would you be to seek treatment to improve any issues you have described now?
  - Where would you seek help with these issues?
- Which of these areas you rated on the PSQI would you want to change?

*How did you find completing the PSQI?*

*Is there anything you think it should have asked about that it didn’t?*

- Is there anything you have tried which helped your sleep? *(how did it help?)*
  - Is there anything anyone else has suggested, or done, which helped (friend, family, healthcare professional, book, internet or TV)? *(how did it help?)*
- Is there anything you have tried / anyone else has tried, that didn’t help?

*Why do you think that wasn’t helpful for you?*

Sleep interventions cards:

- Which ones do you think would be most likely to work?

*For you? For others? And why?*

- Which would be the easiest, and which would be the most difficult to use?

*why?*

- How long would you expect X to take to work?
- How would you judge if X was working?

How likely do you think it is that your sleep will improve in future?

Anything else you want to add?

| **information and advice about sleep**  (e.g. booklet, website, video/DVD, tape) | **advice from a health professional**  (e.g. CPN, care co-ordintor, support worker, OT, psychiatrist, GP, psychologist) |
| --- | --- |
| **reduce use of nicotine**   - reduce smoking / quit smoking - try not to smoke just before bed or in the night | **alternative therapies**   - acupuncture - aromatherapy - herbal remedies - Other___________________________ |
| **cognitive behavioural therapy for insomnia (CBT-i)**   - usually weekly appointments for 6 weeks - cognitive therapy, challenging unhelpful thoughts - tasks to do between sessions (see ‘stimulus control’, ‘relaxation techniques’ and ‘regular routine’) - face-to-face / phone / online - group / individual | **sleep restriction**   - reduce the hours in bed per night to the amount you are actually sleeping - avoid naps - as you are more tired you may fall asleep more quickly - gradually increase the amount of time in bed until you find the optimum amount of time - you might be more tired doing this at first |
| **stimulus control**   - reducing the stimulus before bedtime (e.g. do something quiet, dim the lights, no computer or TV) - avoid laptops, TV, smartphones and other distractions in bed area - associate bedroom with sleep | **regular routine** (train your body clock)   - try to stick to regular getting up times and bed times - try to avoid lying in or staying up later than usual - try to avoid daytime naps - get plenty of natural light in the day |

| **relaxation techniques**   - progressive muscle relaxation - breathing techniques - mindfulness meditation   some people find yoga, or tai chi relaxing  for some people praying relaxes them | **physical activity**   - increase exercise - could include: sports, gym, running, walking, active tasks like housework, active tasks at work - avoid exercise in the 4 hours before bed |
| --- | --- |
| **reduce use of caffeine**  (in coffee, tea, coke/pepsi, energy drinks, chocolate)   - reduce caffeine - don’t drink caffeine as late in the day | **diet**   - try to eat healthily - try to eat regular meals - try not to eat large meals near bedtime - eat foods that help your body with sleep (milk, honey, nuts, bananas, wholegrains) |
| **activity and routine**   - busier routine in the daytime - take on new activities (work/leisure/social) | **talking therapies / psychotherapy / counselling** |
| **prescribed medication** | other________________________________ |
| **vitamin or mineral supplements** | other________________________________ |
