# Supplementary material for: Experiences, perspectives and priorities of people with schizophrenia spectrum disorders regarding sleep disturbance and its treatment: a qualitative study
Source: BMC Psychiatry. 2017 May 2;17:158. doi: 10.1186/s12888-017-1329-8 (PMC5414297; doi:10.1186/s12888-017-1329-8)
Supplement: Supplementary file 2 — Creation of separate Nvivo projects for each case. (DOC 457 kb) [file 12888_2017_1329_MOESM2_ESM.doc]

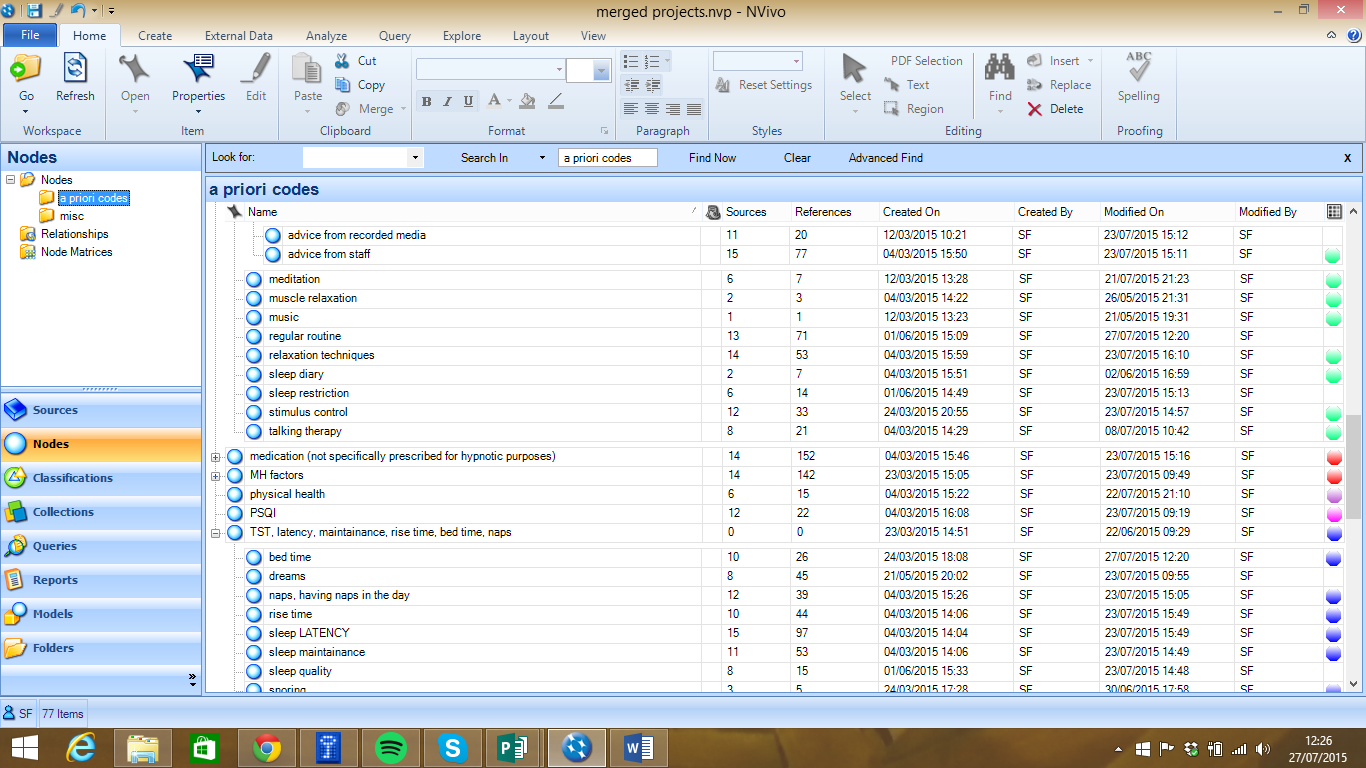
Codes (nodes) created in Nvivo 10 are displayed and available to drag and drop quotations to.


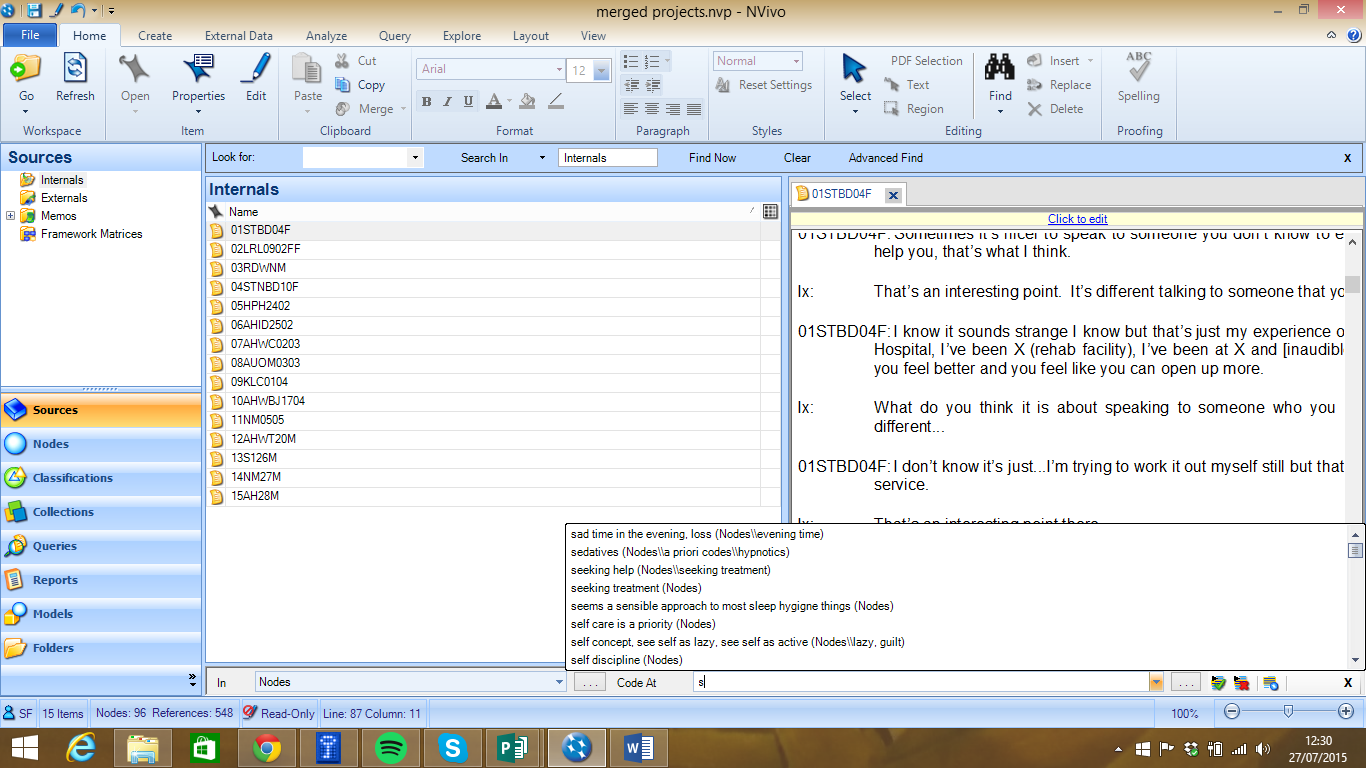


Existing codes are automatically predicted in the ‘code at’ bar when typing the first letters of the name of that code.

To avoid being prompted to interpret new cases using similar codes to preceding cases a new Nvivo project was created for each case, in order to create new interpretive codes. The same a priori codes, to group content by topics were used in each Nvivo project. Projects were merged after case-by-case analysis was complete and interpretive codes compared.

| **Examples of a priori codes which were transferred between cases:** | **Examples of interpretive codes which might be unique to one case:** |
| --- | --- |
| - sleep latency - sleep maintenance - total sleep time - snoring - dreams - diet - exercise - aromatherapy - muscle relaxation | - adjusting medication timings to suit - the world is not a nice place - accepting and recalibrating - spiralling pattern with sleep - is it normal - lazy - using caffeine to counteract medication - searching for a cause - self-discipline |
