# Supplementary material for: Experiences, perspectives and priorities of people with schizophrenia spectrum disorders regarding sleep disturbance and its treatment: a qualitative study
Source: BMC Psychiatry. 2017 May 2;17:158. doi: 10.1186/s12888-017-1329-8 (PMC5414297; doi:10.1186/s12888-017-1329-8)
Supplement: Supplementary file 3 — Additional data excerpts – themes, sub-themes and codes. (DOC 66 kb) [file 12888_2017_1329_MOESM3_ESM.doc]

| **Theme** | **Sub-themes and codes** | **Data excerpts** |
| --- | --- | --- |
| **Sleep priorities: sleep quality and sleep maintenance** | Sleep quality | “Respondent: It's shallow and light and I don't energise the way I should.  Interviewer: Can you tell me more about what that's like to have shallow light sleep?  Respondent: It's like getting into a bath, and it's not full, and you need to bath yourself, there's just not enough clean water to have a proper wash and get the day's dirt off without coming out dirty.” (r02) |
|  | Sleep maintenance is valued | “Interviewer: Can you tell me what’s good sleep for you?  Respondent: Going bed at 10 getting up at 8, not broken.” (r10)  “I would say poor sleep is when you keep waking up.” (r08) |
|  | Sleep latency seen as troubling, like in insomnia without comorbidity | “Well I just can't, just can't sleep, I keep having to turn over, lie on one side, lie on the other side. It just...it's just happened loads of times, you know sleeping, it is one of my problems...” (r13)  “Interviewer: How long might you be [in bed] awake without getting up do you think?  Respondent: Not long, I can’t just be lying in bed being awake  Interviewer: How long is not long for you then.  Respondent: 10 minutes, 15 minutes.” (r10) |
|  | Sleep latency no problem, unlike in insomnia without comorbidity  Not caused by worry | “Interviewer: what's it like falling asleep if you're having a really good night sleep would you say?  Respondent: It's okay falling asleep, yeah, [unintelligible] within about an hour…  Interviewer: Do you think that's a long time, a short time, an OK time?  Respondent: No I think that's a short time.” (r01)  “Interviewer: Are you someone that worries at night?  Respondent: Worries at night. I suppose, I’m generally speaking, a worrier. I don’t worry at night specifically.” (r05) |
| **Loss of normality** | Loss related to diagnosis | “I lost my job because of mental health problems.” (r08)  “…it just hurts, for that reason. Knowing that I used to be normal, I'm not normally more.” (r02)  “As a child I always dreamed of doing certain stuff, and now it’s like I’m an adult and them dreams have put to pass and now I think I’m more reality, if you understand me. Because I can go to bed and dream nice things, I have to put it in perspective that these won’t happen to me […] I’ve got to understand that my dreams are not going to become reality.” (r09) |
|  | Loss regarding sleep | “Because you've got memories of being asleep and feeling better than that you know... So you might, you might feel a bit upset […] you know if you've got pleasure out of, like, your sleep, and that, and you wake up and you're feeling good and all that, you'll just, you'll just remember them, anyway, even subliminally, you know what I mean. But I… I just try not to think too much, because of my problems, my illness and all that.” (r13) |
|  | Hope | “I think that if I work with the doctor and the people at […] that in a couple of year’s time hopefully that my mental health will be a lot better, and so would my sleeping be.” (r15) |
|  | Resignation | “Like I say it all boils down to the way I am.” (r10) |

| **Loss of normality (continued)** | Passive coping strategies  Minimising  Using humour | “There’s much more people out there that have got worse problems than me.” (r15)  “It's not something that I think about all the time.” (r08)  “It depends what you define as oversleeping, I suppose. I’ve looked it up on Google and it says things like, a higher risk of heart attack, diabetes or early death [laughs].” (r05) |
| --- | --- | --- |
|  | Recalibration  Positively re-framing | “Respondent: Yeah I was very lazy […] I'm more active now, my tolerances are a lot more now.  Interviewer: Your tolerances?  Respondent: Yeah.  Interviewer: For going without sleep?  Respondent: Yeah.” (r02) |
|  | Need longer sleep  Recuperation | “I could stay in bed 24/7 now (laughs). I just don’t want to get out of bed. I’ve done it over the past week or two, twice I’ve stayed in bed nearly 24 hours. [...] I just want to get back out there, but it’s just like having a broken leg, you’ve got to fix the broken leg before you can get out to work. So that’s all I want to do is go back to work. […] Basically they’re telling me the brain might be only a small organ and stuff like that, but there’s that much going on in it, it needs a break.” (r09) |
|  | Sleep seen as different  Changes to sleep which are hard to describe | “When you have a shower or something, you feel refreshed and you’re like…ah, that’s great, yea that’s…and all that. People are like that about sleep but I’m not like that.” (r12)  “Interviewer: can you think of a time when you've had good sleep  Respondent: I suppose I can but is going back a bit though  Interviewer: […] what was it like having good sleep?  Respondent: It's a lot different…” (r10) |
| **Knocking yourself out** | Hypnotics, disadvantages of.  Hypnotics, last resort | “I'm not having that, had it once, sleeping tablets but it makes me feel weird. […] You’re not getting a proper, a natural sleep then you, is just false and knock you out” (r06)  “…short term kind of answer, and you’re living on them things and depending on them.” (r09)  “…at one point they had to give me sleeping tablets.” (r11) |
|  | Antipsychotics as a necessary compromise  Antipsychotics as not ideal | “I don't think I've got a sleep problem at the moment, it's a funny one because I don't have a sleep problem because I take medication, but I do have a sleep problem because if I didn't take that medication I know for sure that I wouldn't sleep, so I don't know the answer to that. And what I would like to know is the sleep I have, is it is natural as the sleep someone has without medication? I'd like to know that.” (r08)  “Respondent: Because it's what I do every night anyway, I've just got to go with the realistic one, the easiest option.  Interviewer: The easiest option.  Respondent: Yeah take the medication wind down and get my head down.  Interviewer: Is that what you prefer really?  Respondent: I don't prefer it but it's what I've been doing, but I'm learning to do other things, but when it comes to it, that's my last option.  Interviewer: That's the last option actually, not the first option.  Respondent: Not the first, it's the last one.” (r02) |
| **Knocking yourself out (continued)** | Antipsychotics seen as a sleeping tablet | “Respondent: Because I wouldn't mind, if I could, I wouldn't mind trying sleeping without the medication. Not yet, but I wouldn't mind trying it.  Interviewer: So do you think the medication is, sort of, mostly for your sleep?  Respondent: Well it seems to be…” (r03)  “Respondent:: Quetiapine  Interviewer: is it prescribed for your mood or is it prescribed for your sleep?  Respondent: It’s like half and half.” (r10) |
|  | Natural sleep VS False sleep | “…they knock me out.” (r03)  “Like they say about sleeping tablets, but I wouldn’t want them, because you rely on then then to get sleep, and that’s a false sleep then isn’t it?” (r10) |
|  | Controlled by medication  Reliant on medication | “Yeah I know I just wouldn't sleep so, I rely on what I take. But on the one hand I don't like the idea of being controlled by medication, but if that's what I have to do then that's what I have to do to stay okay.” (r08)  “If I’m being honest, it seems like I’ve got this cycle kind of thing going on, which is good for me on the one hand, it’s keeping things quite regimented, but am I dependent on medicine to actually… That’s what you could say I’m not happy about.” (r04)  “Respondent: I'm into a routine I go to sleep every night. I have tested myself and come away from the medication and it's gone haywire.  Interviewer: Right.  Respondent: Haywire, I've been up for three days.” (r02) |
|  | Efforts to control sleep | “Respondent: Yeah, er yeah I get baths  Interviewer: having a bath, that helps?  Respondent: Yeah. Sometimes a like smoke a little bit of heroin.” (r13)  “I try and force myself to go to sleep…” (r15) |
|  | Uncontrollable sleep | “You know one day I can feel fine, and get up, you know the next day, totally different. Can’t tell what day is going to be the same.” (r10)  “I just have to leave it to God, because there’s nowt you can do really, is there?” (r12)  “Respondent: Does your hair ever stand-up on end on your legs  Interviewer: yeah, yeah I know that feeling  Respondent: My brain says go and I go.” (r02) |

| **Priorities and life goals: daytime functioning** | Benefits of adequate sleep  Impairments from poor sleep | “Well you get more energy don’t you and you get a little bit more willpower to carry on. You reflect on the world and it’s a new start.” (r15)  “My eyes go light, my skin goes pale, I don't eat a lot, I get very driven, I get angry.” (r02) |
| --- | --- | --- |
|  | Vocational goals are contingent on sleep | “Well I don't know because it depends whether I'm working or not. Because just normally I'd be getting up and going to work. […] Hmm, well I don't mind… Well my sleep is okay…for now...” (r01)  “I might find it difficult to get up in the morning after a period of time, do you know what I mean, because having to get up for work every day at the same time, because I'm not in a routine of getting up at the same every day, going to bed at the same time.” (r14) |
|  | Social pressure to appear alert  Social image  Self image | “And I'd like to stay up, you know, with people. Because I have a friend stop at my house sometimes, and I'd like to be able to stop up, you know, and just talk or watch the telly, you know. Because I feel rude going to bed when I've got a friend stopping over.” (r03)  “So I haven’t told anyone about my sleep problems. So I’m quite worried about how people will take it, and how I’ll deal with it.” (r05) |
|  | Increased sleep inertia is a problem | “Because I used to wake up bright and breezy, you know. […] I always had energy in the morning. Well, at the moment, the energy is lacking a bit, when I first get up.” (r03)  “Interviewer: So if there was anything you could change in an ideal world, about sleep, what would it be?  Respondent: The recovery after sleep.  Interviewer: waking up?  Respondent: Yeah  Interviewer: What would be different?  Respondent: Coming-to quicker  Interviewer: How quickly?  Respondent: Half an hour.” (r02) |
|  | Excessive sleep as unacceptable | “It will just bug me something…you’re lazy, you’re lazy, get up, get up, you know, you’re wasting your life away.” (r12)  “…but it's just that I'm not using the energy during the day, so I'm actually needing less and less sleep, erm, and that's not happening you know, I seem to still be sleeping a longer time.” (r01)  “…is it to keep them asleep for the rest of their life or is it to keep them active and let them progress along their life? What should it be? Should there be a balance of keeping people on medication or people off medication.” (r09) |
| **Sleep as an escape: surviving** | Sleep as an escape | “[I nap because]…I don’t wanna wait, because I don’t like being on my own with the voices.” (r15)  “I just don’t want to get out of the dream, even though the dream might be an illusional dream or whatever, it’s just somewhere nice where I feel safe, and I don’t want to be out of that comfort zone, so I just stay in bed. I’m thinking what’s the point in getting up and doing things when I’m in my little comfort zone in bed. If someone needs me  they’ll ring me, so I’ll just stay in bed, and that’s what I’ve done.” (r09) |
| **Attitudes to non-pharmacological intervention: gradual imperceptible effects** | Complex  Seen as a specialist topic | “Respondent: [advice] Mmm.... Maybe a psychologist?  Interviewer: Mmmmhm.... They might be a good source of advice about sleep you think?  Respondent: Possibly yes  Interviewer: What about the other ones [professions on cue card] as well?  Respondent: It depends what their interests were, if they weren't particularly interested in sleep then maybe they won't be so good at giving advice.” (r08) |
|  | Individual | “[breathing techniques] I’ve got no time for stuff like that.” (r12)  “I love saying my prayers […] that does relax me” (r12) |
|  | Learning from experience | “I do know that in terms of relaxing you’ve got to find out what works for you” (r04) |
|  | Whole lifestyle approach | “And taking a kind of, what’s the word, tough love approach, kind of thing. Like making myself … because the word commitment comes out then. And like if I make myself have a commitment to somebody else, and I don’t want to let them down, then that might be a strategy for dealing with it.” (r05) |
|  | Seeing routine as important | “I try to stick to a pattern of going to bed at a certain time, and, because from experience that's important.” (r08) |
|  | Sleep timing unimportant  Sleep timing altered | “…four hours maybe, a good four hours during the day. Which is good, which is good.” (r15)  “I think my body clock is really p***ed up, man [...] It’s gone west. It doesn’t register.” (r12) |
|  | Behavioural change can be challenging | “Respondent: …because I'm not in a routine of getting up at the same every day, going to bed at the same time […]  Interviewer: Do you think it would be hard to get into a routine then?  Respondent: I think it would initially for the first three to six months […] But, I think once you get into a routine of doing something, it just become second nature doesn’t it?” (r14)  “I suppose if I thought I had some support. I don’t think I would do it, just like, 'heck, I might try and see how it goes'. But if I had some support, I probably would try it.” (r03) |
|  | Harmless but ineffective | “[Information and advice] Probably, I don't know if it would make much difference, there's no harm in trying though.” (r06) |
