# Supplementary material for: Experiences, perspectives and priorities of people with schizophrenia spectrum disorders regarding sleep disturbance and its treatment: a qualitative study
Source: BMC Psychiatry. 2017 May 2;17:158. doi: 10.1186/s12888-017-1329-8 (PMC5414297; doi:10.1186/s12888-017-1329-8)
Supplement: Supplementary file 4 — Participant opinions on specific interventions. (DOC 106 kb) [file 12888_2017_1329_MOESM4_ESM.doc]

|  | **Reduce caffeine** | **Exercise** | **Diet** |
| --- | --- | --- | --- |
| r01 | I don't find that it's had and the effect on keeping me awake | Well to be physically tired, more tired, means it's easier to go to sleep at night | - |
| r02 | No I'll be up all night | I like to swim, I like saunas. They're all relaxing | Yea, not just before bed |
| r03 | caffeine does keep you awake, doesn't it. | if you do things in the day, you can tire yourself out, can't you, naturally. | eat regular meals […] I've read about bananas […] can help you sleep |
| r04 | "Caffeine keeps you up" not me. / Just test it […]see if it’s made any difference | [swimming] it used to tranquilise me. | I try not to eat large meals near bed time |
| r05 | I don’t really have much belief in caffeine in tea | I’m sure it works / go walking. I’d be willing to do that. | I wouldn’t say a large meal, no, because I wouldn’t sleep well. |
| r06 | - | well I go to the gym | - |
| r07 | I don’t like drink any tea after seven at night […] trying to cut down | that might help me a little bit. / that’s the one I’m focussing on | maybe I need to sort of like re-think the times that I’m eating |
| r08 | good advice / [not] sensitive to caffeine […]don't notice the difference | I think it would do yes. But I think if people are beyond a certain level of sleeplessness, erm, I think exercise won't make any difference to you | I think that's probably good try not to eat large meals before bedtime |
| r09 | I might take a bottle of Coke to bed it doesn’t disturb my sleeping | - | Does it make any difference? […] No. |
| r10 | I don’t really drink tea or coffee | yeah yeah … (places physical activity card into good idea list) / I’ve done that, exercise | try to eat healthy, do that try to do that |
| r11 | That's a good one, avoiding stimulant drinks. | It does help, yeah. | I can't sleep on an empty stomach […] nothing too heavy |
| r12 | - | I might give it a go but I’m not really keen on it | I think it must [impact on sleep] |
| r13 | I’ve done it loads of times have a brew then gone back to sleep straight after | Physical activities, burn off a few joules […] stretches, yea | - |
| r14 | that can keep me awake | Yes, I do sleep well [after exercise] yes. | - |
| r15 | - | walking round the block and that’s helped me a lot it has | - |

**Key:**

Positive opinion Neutral / conflicting opinions / it depends Negative opinion - Undecided / don’t know / no opinion / not discussed

|  | **Herbal remedies or supplements** | **Aromatherapy** | **Acupuncture** |
| --- | --- | --- | --- |
| r01 | - | - | - |
| r02 | it's working | Yea hot bath, piping. And putting a sweet smell on | I think I'd be so flipping […] responsive to it. |
| r03 | - | I've bought aromatherapy oils […] it's very relaxing | - |
| r04 | - | - | - |
| r05 | - | It’s still a pleasant thing. | - |
| r06 | I don't know | - | - |
| r07 | that could be an option | it never works / I use… lavender […] supposed to be good | I’m a bit scared of needles / I’d be willing to try |
| r08 | maybe they are good I don't know | aromatherapy is good | It didn't really work for me […] I'm sure for some people it does work |
| r09 | - | That’s okay | - |
| r10 | I’m on them | - | I know that it wouldn’t work for me |
| r11 | I think that will help | it can relax you | Good […] because it helps with stress and anxiety |
| r12 | - | - | - |
| r13 | - | - | - |
| r14 | there’s a lot of […] scientific benefits in herbal stuff | I'm not into it. | Acupuncture, I wouldn't. |
| r15 | - | - | - |

**Key:**

Positive opinion Neutral / conflicting opinions / it depends Negative opinion - Undecided / don’t know / no opinion / not discussed

|  | **Relaxation techniques** | **Stimulus control** | **Talking therapy** |
| --- | --- | --- | --- |
| r01 | - | - | - |
| r02 | it's hard to focus on really submerge | […TV in your room?] Yeah it's got to go | - |
| r03 | They're good[…]I use them mainly at night. […]So they do help relax | Because I think it can heighten your senses. […] screen, isn't it. Same with the TV, can't it, as well | - |
| r04 | They work for so many people. They wind me up / find out what works for you | in the bath for half an hour, an hour or whatever, and that’s how I unwind | - |
| r05 | Falling asleep isn’t the problem, it’s the oversleeping. | [computer] not associated with the bedroom in any way. Yea that seems fairly straightforward | I’m on a waiting list to see a psychologist, but I don’t know […] how they might help |
| r06 | …it was okay [was it relaxing?]  Yea [would you apply it to your sleep] No. | - | (shakes head) |
| r07 | I find them quite good, yeah […] I could try them again and see where it goes | I have been given advice but it never works… not watching TV before you go to bed | Maybe, yeah. |
| r08 | breathing techniques probably are very good / [mindfulness] didn't work for me | - | it depends who the therapist was, and what sort of therapist |
| r09 | I suppose they’re good on some occasions. I suppose they’re okay. | I have Classical FM on the radio, turn all the major lights off | - |
| r10 | It’s all right but it wouldn’t help me go to sleep | No. I don’t do any of them in the bed anyway. | I’ve had it [for sleep or for something else?] something else [it didn’t help?] No |
| r11 | Progressive muscle relaxation, that will help. Breathing techniques help. | watch TV in bed [to help sleep] / Yeah, dim the lights, that’ll help | I don't know if that will work […] I'll put that as a no. |
| r12 | I’ve got no time for stuff like that. / I love saying my prayers […] that does relax me. | - | - |
| r13 | Yeah I try things like that yeah, lots of little tricks like that | - | - |
| r14 | I'm not into this new age stuff | I just fall asleep in front of the TV, that's what I do | - |
| r15 | Yeah. Definitely | the environment you sleep in it is important. | I could speak my mind to them […] that might help me sleep |

**Key:**

Positive opinion Neutral / conflicting opinions / it depends Negative opinion - Undecided / don’t know / no opinion / not discussed

|  | **Regular routine** | **Busier daytime routine** | | **Sleep restriction** |
| --- | --- | --- | --- | --- |
| r01 | - | more tired, means it's easier to go to sleep at night | | - |
| r02 | Mmm, I'm not too sure, it depends on the person's make up | - | | - |
| r03 | you get yourself into a routine [Is routine important?] Yes, I think so. | - | | - |
| r04 | routine’s key | having things to do and being busy[…]suits me | | - |
| r05 | I’m sort of a creature of habit really. So, it’s breaking the habits really | - | | that’s trying to induce physical sleepiness, physical tiredness […] I’d be willing to try. |
| r06 | That probably helps a bit yeah | I’m always out […] that one is good yea | | - |
| r07 | I had to go to bed later because I thought well I’m just going to be lying in bed | Probably yeah | | - |
| r08 | I try to stick to a pattern of going to bed at a certain time […] that's important. | I would imagine so | | - |
| r09 | [I need to] get back into a routine of getting up and sorting myself out | - | | [sleep] in the afternoons it’s going to disturb your sleep pattern in the evening |
| r10 | I don’t know, I’ve done that and it doesn’t happen. | I disagree | | [it’s hard] Just staying awake! […] I always go to sleep in the afternoon |
| r11 | I'll put that as a yes. | yeah, because if you keep active, your body's going to be tired […] more prone to sleep | | …your body's going to be tired throughout the day. So I think that would help |
| r12 | I think my body clock is really p***ed up [...] It’s gone west. It doesn’t register. […] that should[n’t] matter, should it, getting up times and going to bed times? | | - | - |
| r13 | - | - | | - |
| r14 | once you get into a routine… [better sleep] that would come if I got a job | [sleep would improve] if I start getting a stimulus…stimulation in the daytime | | It’s probably because […] I've woke up too late in the morning |
| r15 | Probably would help, yeah. I just tend to stay up you know to join in sometimes | Well you need to increase your activity to reduce the stress. | | - |

**Key:**

Positive opinion Neutral / conflicting opinions / it depends Negative opinion - Undecided / don’t know / no opinion / not discussed

|  | **Reduce caffeine** | **Exercise** | **Diet** | **Herbal remedies/ suppleme-nts** | **Aromathe-rapy** | **Acupunct-ure** | **Relaxation techniques** | **Stimulus control** | **Talking therapy** | **Regular routine** | **Busier daytime routine** | **Sleep restriction** |
| --- | --- | --- | --- | --- | --- | --- | --- | --- | --- | --- | --- | --- |
| r01 | **X** | **X** | **-** | **-** | **-** | **-** | **-** | **-** | **-** | **-** | **X** | **-** |
| r02 | **X** | **X** | **X** | **X** | **X** | **X** | **X** | **X** | **-** | **X** | **-** | **-** |
| r03 | **X** | **X** | **X** | **-** | **X** | **-** | **X** | **X** | **-** | **X** | **-** | **-** |
| r04 | **X** | **X** | **X** | **-** | **-** | **-** | **X** | **X** | **-** | **X** | **X** | **-** |
| r05 | **X** | **X** | **X** | **-** | **X** | **-** | **X** | **X** | **X** | **X** | **-** | **X** |
| r06 | **-** | **X** | **-** | **X** | **-** | **-** | **X** | **-** | **X** | **X** | **X** | **-** |
| r07 | **X** | **X** | **X** | **X** | **X** | **X** | **X** | **X** | **X** | **X** | **X** | **-** |
| r08 | **X** | **X** | **X** | **X** | **X** | **X** | **X** | **-** | **X** | **X** | **X** | **-** |
| r09 | **X** | **-** | **X** | **-** | **X** | **-** | **X** | **X** | **-** | **X** | **-** | **X** |
| r10 | **X** | **X** | **X** | **X** | **-** | **X** | **X** | **X** | **X** | **X** | **X** | **X** |
| r11 | **X** | **X** | **X** | **X** | **X** | **X** | **X** | **X** | **X** | **X** | **X** | **X** |
| r12 | **-** | **X** | **X** | **-** | **-** | **-** | **X** | **-** | **-** | **X** | **-** | **-** |
| r13 | **X** | **X** | **-** | **-** | **-** | **-** | **X** | **-** | **-** | **-** | **-** | **-** |
| r14 | **X** | **X** | **-** | **X** | **X** | **X** | **X** | **X** | **-** | **X** | **X** | **X** |
| r15 | **-** | **X** | **-** | **-** | **-** | **-** | **X** | **X** | **X** | **X** | **X** | **-** |

**Key:**

Positive opinion Neutral / conflicting opinions / it depends Negative opinion - Undecided / don’t know / no opinion / not discussed
